# High Sensitivity Plasmonic Sensor Based on Fano Resonance with Inverted U-Shaped Resonator

**DOI:** 10.3390/s21041164

**Published:** 2021-02-07

**Authors:** Gongli Xiao, Yanping Xu, Hongyan Yang, Zetao Ou, Jianyun Chen, Haiou Li, Xingpeng Liu, Lizhen Zeng, Jianqing Li

**Affiliations:** 1 Guangxi Key Laboratory of Precision Navigation Technology and Application, Guilin University of Electronic Technology, Guilin 541004, China; xiaogl.hy@guet.edu.cn (G.X.); 19022304015@mails.guet.edu.cn (Y.X.); 19022202023@mails.guet.edu.cn (Z.O.); 19022202002@mails.guet.edu.cn (J.C.); lihaiou@guet.edu.cn (H.L.); tadyliu@guet.edu.cn (X.L.); 2 Guangxi Key Laboratory of Automatic Detecting Technology and Instruments, Guilin University of Electronic Technology, Guilin 541004, China; 3 School of Electronic Engineering and Automation, Guilin University of Electronic Technology, Guilin 541004, China; 4 Graduate School, Guilin University of Electronic Technology, Guilin 541004, China; zlzh@guet.edu.cn; 5 Guangdong-Hong Kong-Macao Joint Laboratory for Intelligent Micro-Nano Optoelectronic Technology, Foshan University, Foshan 528225, China; jqli@must.edu.mo

**Keywords:** Fano resonance, metal-dielectric-metal, finite element method, plasmonic nanosensor

## Abstract

Herein, we propose a tunable plasmonic sensor with Fano resonators in an inverted U-shaped resonator. By manipulating the sharp asymmetric Fano resonance peaks, a high-sensitivity refractive index sensor can be realized. Using the multimode interference coupled-mode theory and the finite element method, we numerically simulate the influences of geometrical parameters on the plasmonic sensor. Optimizing the structure parameters, we can achieve a high plasmonic sensor with the maximum sensitivity for 840 nm/RIUand figure of merit for 3.9 × 10^5^. The research results provide a reliable theoretical basis for designing high sensitivity to the next generation plasmonic nanosensor.

## 1. Introduction

Surface plasmonic sensing technology is considered one of the most promising plasmonic applications, which have important chemical analysis applications, biological monitoring, and other fields [1,2,3]. As a branch of surface plasmonic research, surface plasmon Fano resonance has become a hot spot in sensing due to its steep asymmetric response spectrum, field enhancement effect, high refractive index sensitivity, and a high figure of merit [4,5]. This Fano resonance is caused by the coupling of the narrowband dark mold and the bright broadband mold in the structure. Unlike the traditional Lorentz line, Fano resonance has sharp asymmetry, which is very sensitive to the surrounding environment’s variation and structural parameters. It can obtain higher sensitivity and a figure of merit, thus in the refractive index, sensing aspect is deeply concerned [6,7]. In 2010, Boris et al. [8] reviewed the Fano resonance in plasmonic nanostructures, metal photonic crystals, and metamaterials, indicating that Fano resonance can be applied to label-free detection [9], filter [10,11], sensor [12,13], slow light device [14,15], optical switch [16], etc. Studies have shown that surface plasmon polaritons (SPP) are the electromagnetic wave produced by the interaction between free electrons and lightwave on the metal surface. The electromagnetic wave propagates along with the interface between the metal and the dielectric and attenuates exponentially in the direction perpendicular to the metal dielectrics interface. [17,18]. Using the unique light properties of surface plasmon breaks the diffraction limit of traditional optics. Optical devices have low loss, small size and long propagation distance, easy integration, and other advantages [19,20]. In recent years, researchers have used the Fano resonance generated by metal nanostructures to conduct a lot of research on their nanostructures’ propagation characteristics and sensing properties. For example, in 2016, Zhang et al. [21] reported a refractive index sensor based on metal-insulator-metal (MIM) waveguide coupled double rectangular cavities. The Fano resonance peak depended on the mode coupling between the double rectangular cavities.

The results show that the maximum sensitivity (S) of 596 nm/RIU, the maximum FOM of less than 10. In 2017, Zhao et al. [22] proposed that the tunable Fano resonance can be realized by coupling an asymmetric structure of a ring resonator on the side of the metal baffle. Changing the ring resonator’s inner radius, the maximum sensitivity, and the ultimate figure of merit can be up to 718 nm/RIU, 4354, respectively. In 2019, Yang et al. [23] proposed a structure consisting of two symmetrical triangular waveguides and a ring split ring resonator cavity (CSRRC). Its design is under a maximum sensitivity of 1500 nm/RIU, the highest FOM is 65.2, but the FOM is not high enough. The above studies have controlled the change of Fano resonance peak by changing the geometric parameters of the structure and the refractive index of the medium. However, most current systems only generate a single Fano resonance and cannot simultaneously obtain high sensitivity and high FOM multi-tuned Fano resonance. Besides, the researchers used experiments to prove that various structures of plasmonic or nanophotonic sensors have high sensitivity. For example, plasma nanorod metamaterials [24], plasma gold mushroom arrays [25], single-phase nanostructures [26], hyperbolic metamaterials [27], nanoporous gold materials [28] and other sensors are proposed. These sensors have high sensitivity, but they need complex preparation methods and expensive optical equipment, so they are difficult to be miniaturized and integrated. At the same time, the above FOM is also low.

In this paper, we design a MIM waveguide structure composed of inverted U-shaped and triangular groove cavities. When TM wave is incident along the MIM waveguide, the SPP generated on the metal surface is coupled to the inverted U-shaped resonator through the F-P cavity, thus causing sharp asymmetry Fano resonance. The numerical simulation is then performed using the finite element method (FEM) to research the impact of geometric parameters and dielectric refractive index on the transmission spectrum, which realizes the tuning of multiple Fano resonance peaks. By optimizing this structure’s parameters, the results show that high sensitivity and a figure of merit can be achieved, so the structure can be used to design multi-channel nanosensor devices.

## 2. Model and Theoretical Analysis

**Figure 1:**
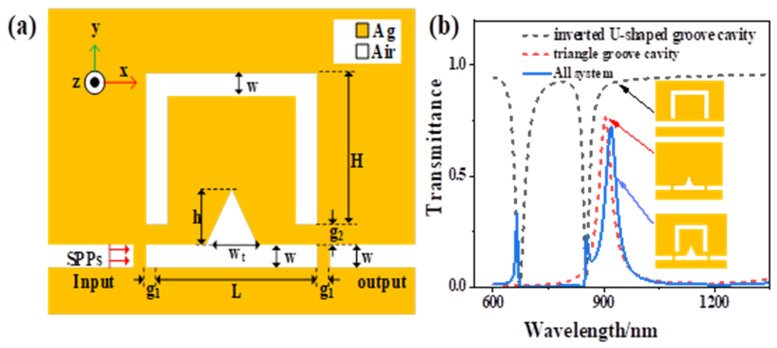
A plasmonic sensor and its transmission spectrum (**a**) Two-dimensional (2D) structure diagram; (**b**) Transmission spectrum of three different structures.

The plasmonic sensor consists of an inverted U-shaped resonant cavity and a triangular groove cavity; the schematic structural diagram is shown in Figure 1a. The yellow part represents metallic silver ($\varepsilon_{m}$); the white part represents the air ($\varepsilon_{d}=1$). As for geometrical parameters include the height of the inverted U-shaped resonant cavity H = 240 nm, the width of the triangular groove cavity $w_{t}=100\;m$, its height h = 100 nm, the length of the groove cavity L = 500 nm, the coupling distance between the input/output waveguide and the triangular groove cavity $g_{1}=10\;\mathrm{nm}$, the coupling distance between the inverted U-shaped resonant cavity and the triangular groove cavity $g_{2}=12\;\mathrm{nm}$. To ensure that only the TM mode is propagated in the MIM waveguide, the width of the straight waveguide w is fixed to 50 nm. The FEM calculates the optical response feature and steady magnetic field distribution of the structure. The scattering boundary is using as the boundary condition. The light source incident at the left port and output at the right port, solve the partial differential equation and carry out simulation calculation to obtain the transmission spectrum. The proposed structure is simple. Firstly, the metal silver layer was deposited on the silicon substrate by chemical vapor deposition. Then, the MIM waveguide and resonant cavity were etched by electron beam etching. The measured medium was injected into the waveguide resonant cavity by capillary attraction and finally sealed with high-temperature resistant sealant. The relative dielectric constant of the silver can be expressed by the Drude model [19]:

(1) $$\varepsilon_{m}\left(\omega \right)=\varepsilon_{\infty }-\frac{\omega_{p}^{2}}{\omega^{2}+i\omega \gamma }$$

Here, $\varepsilon_{\infty }=3.7$, $\omega_{p}=9.1\;\mathrm{eV}$, γ=0.018 eV, $\varepsilon_{\infty }$ is an infinite dielectric constant, $\omega_{P}$ is the plasmonic oscillation frequency, ω is the frequency of electromagnetic radiation (incident light), i is an imaginary unit, γ is the characteristic collision frequency. When there is no triangular cavity, the transmission spectrum forms form a narrow resonance peak, as shown by the black dotted line in Figure 1b. When there is no inverted U-shaped resonator, the transmission spectrum forms a broad resonance peak, as shown by the red dotted line in Figure 1b. When the waveguide with F-P cavity is coupled with an inverted U-shaped cavity, a narrow asymmetric Fano resonance will be produced, as shown by the blue line in Figure 1b. The standing wave theory can be used to determine the resonance conditions of the F-P cavity and the inverted U-shaped resonator, as shown in the following formula [29]:

(2) $$\lambda =\frac{2Re\left(n_{eff}\right)Lp}{n-\varphi /2\pi }n=1,2,3\ldots$$

Here, *n* represents the order of standing wave resonance, *Lp* is the resonator’s perimeter, and φ is the phase change caused by SPP reflection in the MIM waveguide. $Re\left(n_{eff}\right)$ is the real part of the effective refractive index ($n_{eff}$), $n_{eff}$ can be expressed as:

(3) $$n_{eff}=\sqrt{\left[\varepsilon_{m}+{\left(\frac{k}{k_{0}}\right)}^{2}\right]}$$

Here, k=2π/λ is the wave vector in the waveguide.

The Fano resonance phenomenon is analyzed based on the coupled-mode theory, the amplitudes of surface plasmons generated at the input and output ports are expressed as $S_{1+}$, $S_{1-}$, $S_{2+}$, $S_{2-}$ when the light wave with frequency ω is transmitted into the system only at the input port, then $S_{2+}=0$. $\tau_{n_{0}}$ is the decay time of the internal loss of the Fabry-Perot (F-P) resonator and the inverted U-shaped resonator.$\tau_{n1}$, $\tau_{n2}$ is the attenuation time of the coupling between the resonant cavity and the input/output waveguide, respectively. The expression of amplitude variation with time is as follows [30]:

(4) $$\frac{dA_{n}}{dt}=\left(-j\omega n-\frac{1}{\tau_{n0}}-\frac{1}{\tau_{n1}}-\frac{1}{\tau_{n2}}\right)A_{n}+k_{n1}S_{n,1+}+k_{n2}S_{n,2+}$$

(5) $$S_{1-}=-S_{1+}+\sum_{n}k_{n1}^{*}A_{n},k_{n1}=\sqrt{\frac{2}{\tau_{n1}}}e^{j\theta n1}$$

(6) $$S_{2-}=-S_{2+}+\sum_{n}k_{n2}^{*}A_{n},k_{n2}=\sqrt{\frac{2}{\tau_{n2}}}e^{j\left(\theta n2-\phi n\right)}$$

(7) $$S_{n,1+}=\gamma_{n1}e^{j\varphi n1}S_{1+},S_{n,1+}=\gamma_{n2}e^{j\varphi n2}S_{2+}$$

(8) $$\varphi_{n}=\varphi_{n1}+\phi_{n}+\theta_{n1}-\theta_{n2}$$

where $\omega_{n}$ is the resonant frequency of each resonant cavity, $k_{ni}(i=1,2)$ the coupling coefficient between each resonant cavity; $\theta_{ni}(i=1,2)$ the coupling phase between the resonator and the input-output waveguide; $\gamma_{n1}e^{j\varphi n1},\gamma_{n2}e^{j\varphi n2}$ the standard coefficient (where $\gamma_{n1}=\gamma_{n2}=1$); $\varphi_{n}$ the total coupling phase difference of each resonant mode.

When the waveguide has the same width and are symmetrically distributed on both sides of the resonant cavity, we can get $\tau_{n}=\tau_{n1}=\tau_{n2}$, $\theta_{n1}=\theta_{n2}$. According to the previous calculation formula, the transmittance is obtained as follows:

(9) $$T={\left|\frac{S_{2-}}{S_{1+}}\right|}^{2}={\left|\sum_{n}\frac{2\gamma_{n1}e^{j\varphi n}}{-j\left(\omega -\omega_{n}\right)\tau_{n}+2+\frac{\tau_{n}}{\tau_{no}}}\right|}^{2}$$

## 3. Results and Discussion

**Figure 2:**
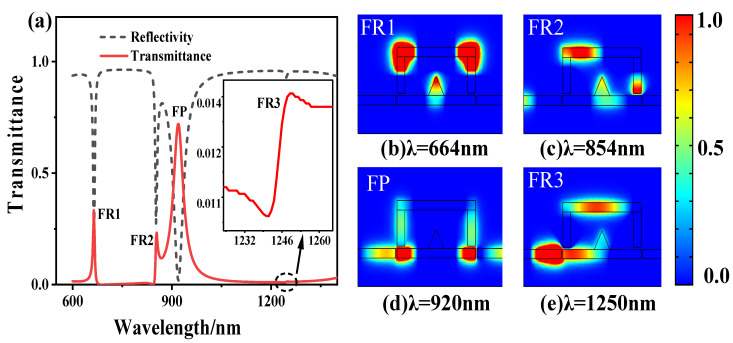
Transmission spectrum and magnetic field intensity distribution of plasmonic sensor (**a**) Reflection and transmission spectrum (**b**–**e**) the magnetic field intensity distribution of FR1, FR2, FP, and FR3.

The reflection and transmission spectrum obtained from a plasmonic sensor is shown in Figure 2a. At the wavelengths of 664, 854, and 1250 nm, there are three sharp asymmetric Fano resonance peaks (denoted as FR1, FR2, and FR3) in the transmission spectrum, and their transmittance is 0.33, 0.23 and 0.0136, respectively. At the wavelengths of 920 nm, a linear symmetrical non-sharp resonance peak (named FP) appeared in the transmission spectrum, and its transmittances were 0.72. Next, we study the characteristics of the Fano resonance peaks of the sensor and analyze the magnetic field intensity distribution of the resonance peaks (FR1, FR2, FR3, FP), as shown in Figure 2b–e.

We can conclude from Figure 2b,c, magnetic field intensity of FR1 and FR2 resonance peaks mostly concentrate in the inverted U-shaped resonant cavity at λ = 664 nm, λ = 854 nm, and only a small amount of energy is distributed in the triangular groove cavity. It can be found that when λ = 664 nm, the magnetic field intensity at the two cavities coupling is in the same direction. We can conclude from Figure 2d, the magnetic field intensity of FP1 at λ = 920 nm. We can see from the figure that most of the energy are concentrated in the input-output waveguide and only a small amount of energy is coupled to the inverted U-shaped cavity. As shown in Figure 2e, the magnetic field intensity of FP1 resonance peak at λ = 1250 nm. We can see from the figure that most of the magnetic field energy is distributed in the left input waveguide and a few in the inverted U-shaped cavity, for the reason of relatively low transmittance. In FR1, FR2, and FR3, the antinodes are distributed in a large proportion in the inverted U-shaped resonator, which has a strong magnetic field distribution. The energy in the resonant cavity is difficult to be transmitted to the waveguide. As a result, SPP is not output to the output waveguide, so Fano resonance occurs, and the transmittance is low. The antinode of the FP distributes considerable energy in the MIM waveguide, and it is difficult for SPP to couple into the inverted U-shaped cavity, so no Fano resonance peak is generated.

**Figure 3:**
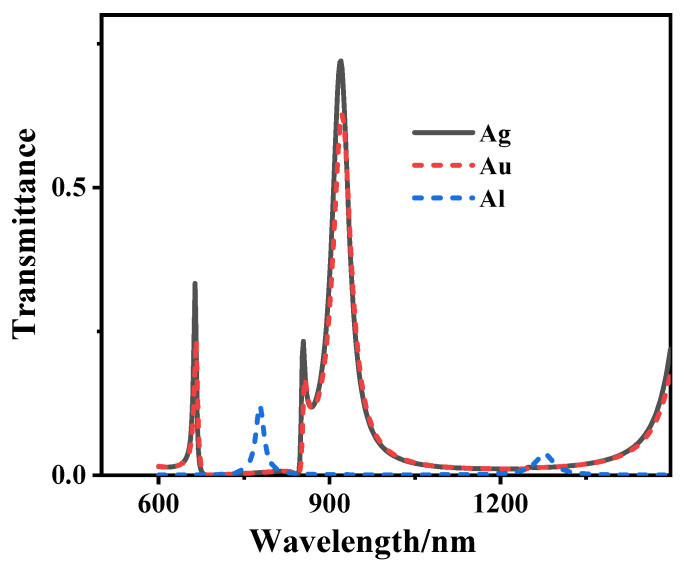
Transmission spectra of Ag, Au, Al.

**Figure 4:**
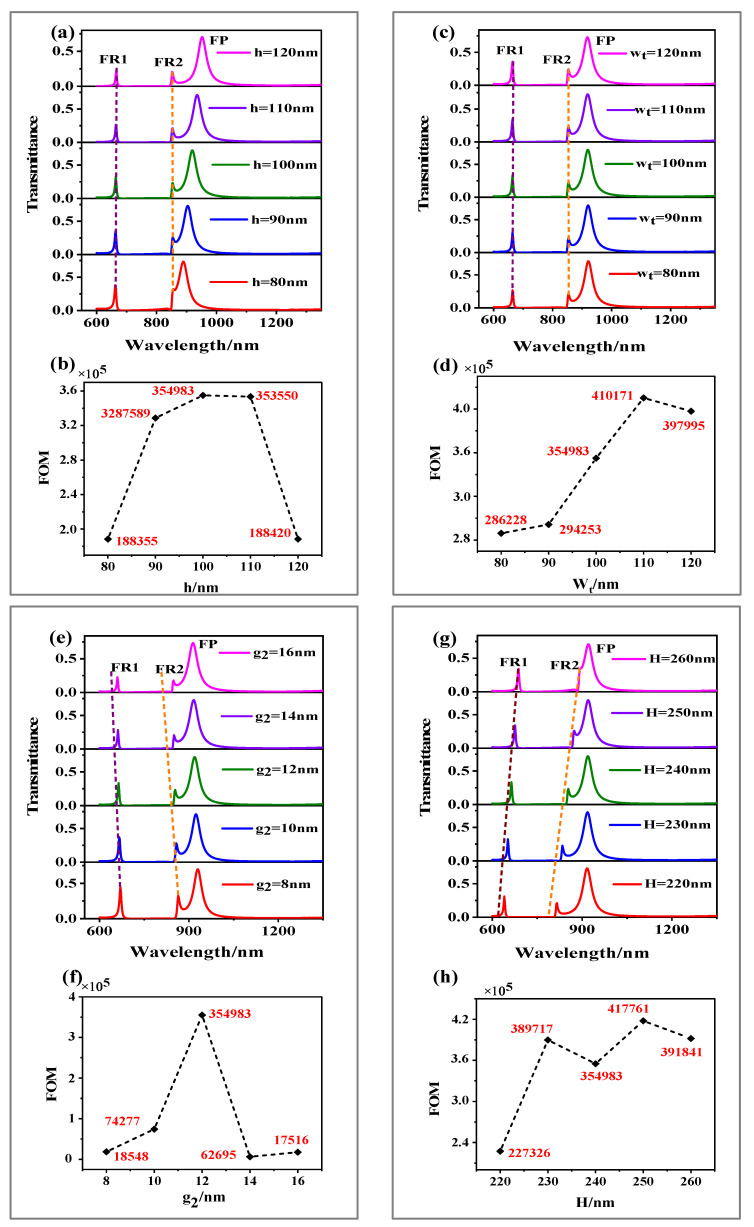
The effect of geometric parameters on the transmission spectrum (**a**) h, (**c**) $w_{t}$, (**e**) $g_{2}$ and (**g**) H as a function of the wavelength; FOM as a function of (**b**) h, (**d**) $w_{t}$, (**f**) $g_{2}$ and (**h**) H.

Firstly, the influence of common metals such as gold (Au), silver (Ag), and aluminum (Al) on the transmission spectrum of the structure is studied. As shown in Figure 3, it can be seen that the transmittance of Ag is higher than that of Au and Al, so it can be concluded that the loss of Ag is the least. Based on this, we use Ag in this structure. We study the optical response of the Fano resonance by changing the parameters of the structure. First, we peer the influence of the height (h) of the triangular cavity on the transmission spectrum. When other parameters remain unchanged, h is scanned from 80 to 120 nm with a scanning interval of 10 nm. The transmission spectrum is shown in Figure 4a. The FOM as functions of h in Figure 4b. We can see from the picture that with the increase of h, the transmittance of FR1 and FR2 decreased and the line type of Fano resonance changed. Under the condition that other parameters remain unchanged, we study the effect of the width ($w_{t}$) of the triangular cavity on the transmission spectrum. When $w_{t}$ is scanned from 80 to 120 nm with a scanning interval of 10 nm, its transmission spectrum is shown in Figure 4c, FOM as functions of $w_{t}$ in Figure 4d. We can see from the picture that with the increase of $w_{t}$ the transmittance of FR1, FR2 increases, and the line type of Fano resonance changes. The above changes are mainly because the wavelength of the Fano resonant peaks is close to the dark mode’s resonant wavelength, which is primarily affected by the dark mode. In contrast, the change of the triangular cavity mainly affects the resonant wavelength of FP.

Then, the effect of the coupling distance ($g_{2}$) between the inverted U-shaped cavity and the triangular groove cavity on the optical properties of Fano resonance is under our research. When other parameters remain unchanged, $g_{2}$ is scanned from 8 to 16 nm with a scanning interval of 2 nm, its transmission spectrum is shown in Figure 4e, FOM as functions of $g_{2}$ in Figure 4f. It can be observed that with the increase of $g_{2}$, the transmittance of FR1 and FR2 decreases, Fano resonance peak appear linear blue shift phenomenon and the line type of Fano resonance changed. Due to the increasing value of $g_{2}$, the coupling strength between the inverted U-shaped resonator and the triangular groove cavity is weakened. Finally, under the condition that other parameters remain unchanged, the height (H) of the inverted U-shaped resonator is increased from 220 to 260 nm, performing a parameter scan with an interval of 10 nm. The transmission spectrum is shown in Figure 4g. The FOM as functions of H in Figure 4h. We can see that the transmittance of FR1 and FR2 increases and shows an apparent linear redshift, and the Fano resonance peak line shape also changes. This is because the height of the inverted U-shaped resonator is increasing, which leads to an increasing in Lp. According to Formula (2), it can be obtained that the resonance wavelength also moves in the long-wavelength direction.

**Figure 5:**
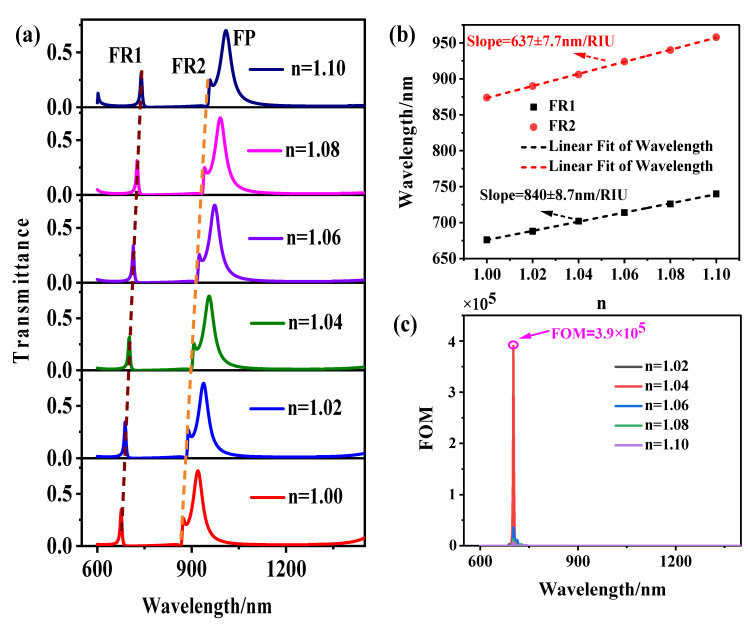
The effect of different n on the sensing performance (**a**) The influence of varying *n* on the transmission spectrum (**b**) Fitting curve of resonance wavelength and refractive index *n* (**c**) The FOM distribution with the wavelength of the structure.

Finally, we discuss the sensor’s characteristics, in which sensitivity (*S*) is a crucial parameter to evaluate the sensor’s attributes. It can be expressed as: S=dλ/dn(nm/RIU) [22], where dn is the change of refractive index, dλ is the change of the resonance wavelength. Changing the white part of the structure in Figure 1a is changing the n of the medium. When the refractive index increases from 1.00 to 1.10 and the scanning interval of 0.02, the transmission spectrum is shown in Figure 5a. It can be seen from the figure that there is a noticeable linear redshift in the resonance wavelength with the increase of *n*. Figure 5b describes the relationship between the *n* and the resonance wavelength. The maximum *S* of the structure is 840 nm/RIU.

Moreover, the figure of merit (FOM) is another critical feature for evaluating sensor characteristics. It can be expressed as: FOM=ΔT/TΔn [31], where T is the transmittance of the system, ΔT/Δn indicates the change of transmittance caused by the transformation of the refractive index of the system in the wavelength range. The FOM distribution of the wavelength of the structure is shown in Figure 5c. Due to the sharpness of the Fano resonance peaks, according to the formula in the figure of merit, it is calculated that when λ = 702 nm, the structure can obtain the maximum figure of merit (FOM) of $3.9\times 10^{5}$. Therefore, compared with previous technologies, our design has greater sensitivity and the highest FOM, as shown in Table 1.

**Figure 6:**
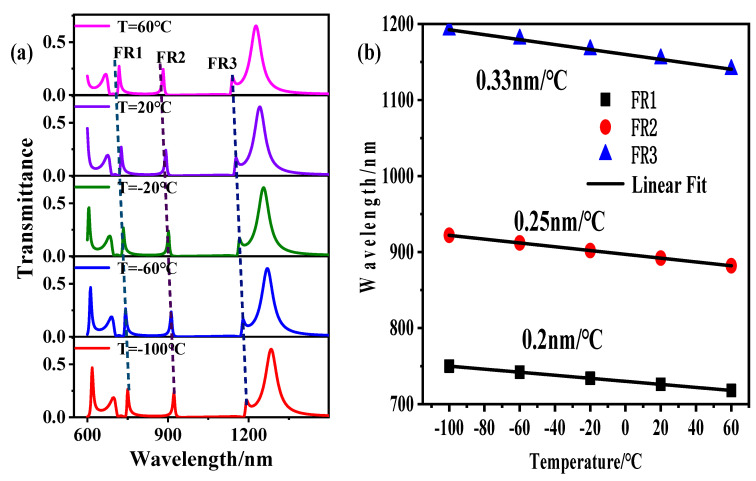
(**a**) Transmission spectra at different ambient temperatures T. (**b**) Linear curve of ambient temperature T and resonance wavelength.

Then, the medium of the structure is filled with ethanol material to realize the temperature sensor. The refractive index of ethanol can be defined as [39]:

(10) $$n=n_{0}-dn∕dT\left(T-T_{0}\right)$$

where, T_0_ is the ambient temperature at 20 °C, T is the ambient temperature, $n_{0}=1.36084$, $dn∕dT=3.94\times 10^{-4}$. Figure 6a shows the transmission spectra at different ambient temperatures. It can be seen from the figure that the resonance wavelength shows a blueshift with the increase of T. According to the formula of ethanol refractive index, with the rise of T, the refractive index n of ethanol significantly decreases. Figure 6b is the linear curve of the relationship between the ambient temperature T and the resonance wavelength. It can be seen that the temperature sensitivities of the three Fano resonances are 0.2 nm/°C, 0.25 nm/°C, 0.33 nm/°C, respectively.

**Figure 7:**
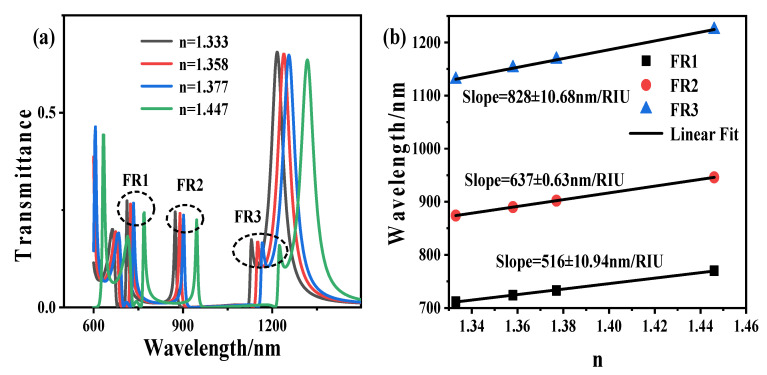
(**a**)The transmission spectra of refractive indices of different liquids. (**b**) The linear curves of the different refractive index and resonance wavelengths.

Finally, to verify that the structure is used for the biosensor, we studied water (*n* = 1.33), acetone (*n* = 1.358), 2-propanol (*n* = 1.377), and chloroform (*n* = 1.446) as the background materials. Figure 7a shows the transmission spectra of the refractive index of different liquids, and Figure 7b shows the linear curves of various refractive index and resonance wavelengths. It can be seen from the figure that the sensitivities of the three Fano formants are 516 nm/RIU, 637 nm/RIU, and 828 nm/RIU, respectively. Through the verification of different liquid refractive index, it is shown that this structure has a potential application in high-sensitivity nanosensors.

## 4. Conclusions

This report designs a MIM waveguide structure, which has a triangular slot cavity between the input and output waveguides, which is coupled with the inverted U-shaped resonator. The FEM is utilized to simulate Fano’s optical characteristics and research the impact of the geometric structure change. The phenomenon of multiple Fano resonance is explained by multimode interference coupled-mode theory. By changing the inverted U-shaped cavity’s height, the coupling distance between the inverted U-shaped cavity and triangular groove cavity, and the height and width of the triangular cavity. It can achieve the change of transmittance and the shift and line type change of Fano resonance peaks, and it has better sensing characteristics. The maximum sensitivity is 840 nm/RIU, and the highest figure of merit is through simulation calculation. It shows that the structure has a broad application prospect in designing plasmonic nanosensors. The sensing properties can also be realized in another style, assuming the nanostructure used to study the polarization state. By investigating these parameters, such as polarization rotation, ellipticity, and so on, we can understand polarization-based nanostructures to achieve high sensitivity. Therefore, we can consider using the polarization state to realize plasmonics nanosensors.

## Figures and Tables

**Table 1:** A comparison of the sensing characteristics of this work with previous work.

Reference	Year	S (nm/RIU)	FOM	Structure
[21]	2016	596	7.5	Double rectangular cavity
[22]	2017	718	4354	Asymmetric ring cavity
[31]	2014	600	650	Rectangular cavity
[32]	2016	600	3803	Two rectangular cavity
[33]	2017	750	68.3	Trapezoid cavity
[34]	2018	497.8	480	Plasmonic metasurface
[35]	2018	680	8.68	T shaped cavity
[36]	2018	350	15	Hybrid Metasurface
[37]	2019	780	1.56 × 10^5^	M-type resonant cavity
[38]	2020	540	101.3	Elliptical cavity
This work	2020	840	3.9 × 10^5^	Inverted U cavity

## Data Availability

Data sharing not applicable.
